# Progress in Rhinology

**Published:** 1895-11-02

**Authors:** 


### Progress in Rhinology.

**The Vascular Mechanism of the Nasal Mucous Membrane, and its Relations to Certain Pathological Processes.**[4]—Jonathan Wright, after summarising the arrangement of the vessels as given by Zucker Kandl, as follows, describes the chief provisions for regulating the supply of blood in the nasal mucous membrane. The larger arterioles, well supplied with muscular coats, lie in the deepest layers, close to the bone. They give off branches which supply by a network of capillaries the periosteum, the glands, and the epithelium. These capillaries are collected into the veins, which dilate into venous sinuses, the larger lacunæ of which are the deeper, the superficial lacunæ or cortical network communicating with them. These lacunæ again empty into the veins which accompany the primary arterioles into the periostial layer." The mechanism which acts upon the deep network of erectile bodies, and also as a supply and escape valve to the cavernous sinuses, depends upon the anatomical relationship of the vein to its companion artery and surroundings. The radical arteries and veins pass through various bony canals into the nose. The artery will evidently compress, when dilated, its accompanying vein against the bony walls, thus letting in more blood, and limiting the outflow. Contraction of the artery acts in the inverse sense. It is an unsettled question whether the artery and vein dilate and contract synchronously; but, supposing they do, the contraction of the muscular coat of the vein would prevent undue engorgement of that vessel (and the consequent too complete emptying of the sinus). Another element in the mechanism of the blood supply is one said to be commonest in the mucosa of the septum, where the density of the muscle fibres in the venous walls is much less marked. Here the veins are compressed between the parallel fibres of the periosteal layer, and the elastic fibres and glands external to it. The mouths of the gland ducts are opened and closed by the contraction and distension respectively of a special network of small veins surrounding them, and hence in an acute coryza, the following is briefly the sequence of events. (1) Vascular engorgement of the erectile bodies leading to nasal occlusion. (2) Watery secretion by transudation directly from the blood-vessel through the areolar tissue and surface epithelium. (3) Collapse of venous sinuses by contraction of the smooth muscle fibres of their walls, and the elastic fibres of the stroma; opening of the gateways of the radical veins by contraction of the encroaching arterioles, and pouring out of mucous secretion by reason of the subsidence of the engorgement of the venous network surrounding the mouths of the glands. Other points in the pathology of hypertrophic and atrophic rhinitis are also touched upon in this paper.

**A Case of Crest on the Septum Narium.**[5]—In this case, described by Guye, a sharp-edged shelf on the septum had in part contracted union with the inferior turbinate—the result of an injury. The spur and narrowing recurred twice after removal, as the septum continued to bulge more and more towards the affected side (the patient was only eighteen). Accordingly, recourse was had to dilatation with india-rubber tubes in the "orthopædic fashion." Tubes of gradually increasing thickness were introduced (6, 8, 10, and 12 m.m.), and retained for all but two hours during the day. At present, the patient has for half a year worn a tube of eleven millimetres in thickness and ten centimetres in length daily for one or two hours. The headache and aprosexia, which clung to him so long as his nose was obstructed, have completely disappeared. It is intended to leave the tube in until the patient attains his full stature and development. The tubes are long enough to reach the posterior wall of the pharynx; when the latter is irritated the tube is drawn out a short distance.

**A New Snare.**—An universal snare has been invented by Dr. Greville Macdonald.[6] It combines the principle of a Jarvis instrument with that of a Morell Mackenzie, and consists of two separate actions, either of which can be used in sequence to the other as occasion may require. These are a trigger and cog-wheel arrangement for the first finger and a set of spokes worked by the thumb, on turning which to the left an inner barrel carrying the looped wire is withdrawn from an outer. Thus, in removing turbinal hypertrophies, the tendency to hæmorrhage is avoided by the slowness of the process. This instrument is so strong that bone and the toughest of fibromata can be taken off without breaking the wire. The latter can be drawn out again ready for action with the greatest ease by removing a collar attached to the outer barrel, and liberating the inner and distal one. The tendency to breaking of the loop after a few operations is obviated, because the noose is not withdrawn within the barrel, but against a rounded extremity, so that the same wire can be used any number of times; further, by turning the end barrel, the loop may be made to present either a horizontal or vertical position. The snare is made by Messrs. Mayer and Meltzer.

[4] Amer. Jnal. of Med. Sci., May, 1895. [5] Monatsch. f. Ohrenheil Kunde, 1894. [6] Lancet, Vol. II., 1894, p. 1287.

